# A Review of the Potential Therapeutic Benefits of Quercetin for Uterine-Related Conditions

**DOI:** 10.3390/biomedicines14061205

**Published:** 2026-05-27

**Authors:** Michael A. Leone, Georgia Kurman, Madeline Bright, Peter K. Gregersen, Christine N. Metz

**Affiliations:** 1 School of Health Sciences, Hofstra University, Hempstead, NY 11549, USA; mleone14@pride.hofstra.edu; 2 School of Medicine, Donald and Barbara Zucker School of Medicine at Hofstra/Northwell, Hempstead, NY 11549, USA; gkurman1@pride.hofstra.edu (G.K.); mbright1@northwell.edu (M.B.); 3 Institute of Molecular Medicine, Feinstein Institutes, Northwell Health, Manhasset, NY 11030, USA; 4 Department of Medicine, Donald and Barbara Zucker School of Medicine at Hofstra/Northwell, Hempstead, NY 11549, USA; 5 Department of OB/GYN, Donald and Barbara Zucker School of Medicine at Hofstra/Northwell, Hempstead, NY 11549, USA

**Keywords:** adenomyosis, chronic endometritis, endometrial cancer, endometriosis, endometrium, flavonoids, quercetin, polycystic ovary disease, uterine fibroids, uterine health

## Abstract

Quercetin is a naturally occurring flavonoid found in fruits, vegetables, and teas that is widely available as a dietary supplement. Numerous studies have investigated quercetin’s therapeutic potential across a broad range of diseases and conditions. Collectively, these studies reveal its anti-inflammatory, antioxidant, anti-proliferative, anti-cancer, anti-fibrotic, antibacterial, endocrine-modulating, and senolytic properties, establishing quercetin as a polypharmacologic agent with diverse biological activities. This review describes quercetin’s biochemical properties, bioavailability, and proposed mechanisms of action. It highlights the unique characteristics of the human uterus vs. other species and evaluates published evidence from pre-clinical and clinical studies supporting quercetin’s pleiotropic effects and potential therapeutic benefits for six uterine-related conditions: endometrial cancer, endometriosis, adenomyosis, uterine infections, uterine fibroids, and polycystic ovary syndrome (PCOS). The findings support that quercetin targets multiple endometrial and other uterine cell types and may attenuate key pathological processes relevant to uterine disease. However, robust human clinical evidence supporting quercetin’s efficacy is generally lacking. Critical knowledge gaps and translational barriers to advancing quercetin from a ‘promising preclinical candidate’ into an ‘evidence-based therapeutic’ for improving uterine health are discussed.

## 1. Introduction

Although nearly half of the world’s population has a uterus, this vital organ receives surprisingly little research attention, especially for health issues unrelated to pregnancy. While the uterus plays key roles in menstruation, fertility, and pregnancy, it is affected by several complex conditions, including endometrial cancer, endometriosis, adenomyosis, uterine infections, uterine fibroids, and polycystic ovary syndrome (PCOS). The pathogenesis of most of these conditions is not well understood. Once diagnosed, patients often have few effective treatment options, many of which are poorly tolerated. The goal of this review is to examine and evaluate pre-clinical and clinical evidence supporting the potential utility of quercetin for treating various uterine health conditions and provide new insights for future studies. Quercetin is a naturally occurring flavonoid found in various plant-based foods. It has also been developed into an over-the-counter supplement. As a polyfunctional agent, quercetin exerts antioxidant, anti-proliferative, anti-inflammatory, anti-cancer, anti-fibrosis, antimicrobial, and senolytic effects. Curiously, few clinical trials investigating quercetin for women’s health, particularly uterine conditions, have been published or posted on clinicaltrials.gov [1], indicating a significant translational gap.

Methods: References for this review were identified through a search of the PubMed, Embase, and Scopus databases and the Google Scholar web search engine. No date or geographical restrictions were applied to ensure comprehensive coverage of the literature. Primary research articles, reviews, and in vitro, animal, and clinical studies were considered. Conference abstracts without full texts were excluded. Search terms were developed based on this review’s population, concept, and context. Terms included a combination of controlled vocabulary (Medical Subject Headings [MeSH] for PubMed, Emtree for Embase, and Scopus INDEXTERMS) and free-text keywords. Keywords were grouped into categories representing population (female), concept (quercetin), and context (uterine health). Boolean operators (AND, OR), quotations, field tags, and proximity operators were used to combine terms appropriately within each database’s syntax. To ensure comprehensive coverage, the search was supplemented by hand-searching the references of selected studies to identify additional relevant publications for review. The databases were searched iteratively, and the final search was conducted on 5 June 2025. The team continued to search PubMed for recently published related articles.

## 2. Quercetin: Background and Proposed Health Benefits

**Figure 1:**
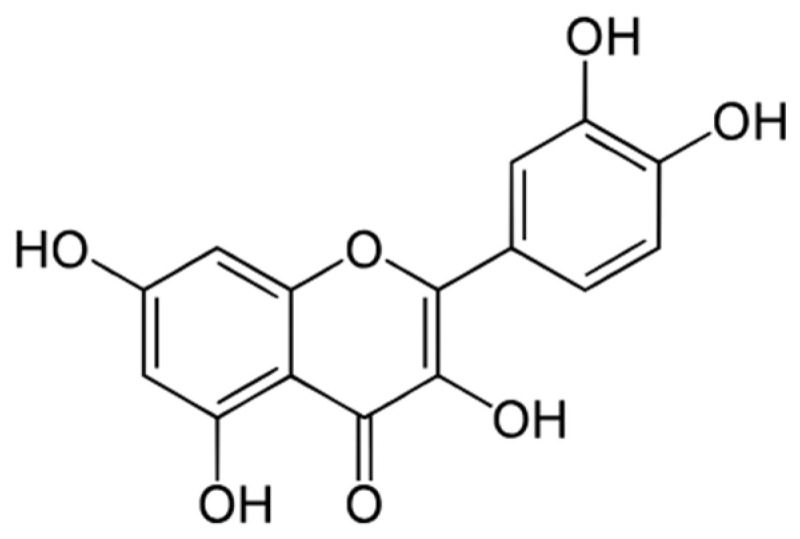
Chemical structure of quercetin.

Quercetin (3,5,7,3′,4′-pentahydroxyflavone), well-known for its diverse bioactivities (e.g., antioxidant, anti-proliferative, anti-inflammatory, senolytic, antimicrobial) [2], is a naturally occurring polyphenolic compound that is part of the flavonoid family. Flavonoids are mainly synthesized by plants and include multiple subtypes, such as anthocyanidins, flavonols, flavanones, flavan-3-ols, flavanonols, flavones, and isoflavones. Structurally, quercetin contains multiple hydroxyl groups attached to its flavonol backbone (Figure 1), which contribute to its redox activity and capacity to interact with diverse targets (e.g., cytochrome P450 enzymes, kinases, drug transporters, and various signaling pathways) [3].

Quercetin concentrations in various plant-based foods vary depending on the plant type, plant component (leaves, stems, flowers, fruit), storage conditions, and preparation methods [4]. Plants containing the highest concentrations of quercetin include capers, rocket (arugula), dill, coriander, and fennel [4]. High concentrations of quercetin are also found in fruits and vegetables, such as red onions, blueberries, apples, okra, and leafy greens, as well as teas [5]. The average daily intake of quercetin is estimated to be tens of milligrams to hundreds of milligrams per day from whole foods alone, depending on the foods consumed [6]. However, this level of quercetin consumption may not deliver its proposed health benefits due to poor absorption [5,6,7].

Quercetin is also consumed as a dietary supplement. Supplemental forms of quercetin are typically in the aglycone form, mostly provided as oral capsules or tablets. Oral bioavailability of most quercetin supplements is low due to poor water solubility (2–10 mg/L for purified quercetin and <10 mg/L for the aglycone form), extensive first-pass metabolism, and fast elimination, which has led to the development of formulations that enhance absorption [8,9].

Beyond its use as a dietary supplement, quercetin is increasingly recognized as a polypharmacological agent due to its ability to interact with multiple molecular targets and signaling pathways [3]. Based on quercetin’s chemical structure, diverse biological properties, and low risk-to-benefit ratio, it has been investigated for disease treatment and prevention in pre-clinical and clinical studies. The results of these studies support quercetin’s anti-inflammatory [4,10,11,12,13], antioxidant [14,15], anti-proliferative [16,17,18,19,20,21,22], anti-migratory [21,23,24,25], antimicrobial [26], anti-fibrotic, and senolytic effects [22,23,27,28,29,30], as well as its endocrine-regulating and metabolic effects [10,31,32,33,34].

These findings, together with its safety profile and negligible toxicity in pre-clinical and early-phase clinical studies [35,36], position quercetin as a multi-target flavonoid compound with potential therapeutic effects across many uterine conditions and pathologies.

## 3. The Uterus and Uterine Health

### 3.1. The Human Uterus and Other Subtypes of Uteruses

The uterus is a major organ of the female reproductive tract, and its role in reproductive-age individuals is complex and multifaceted. The uterus, with input from the ovaries, hypothalamus, and pituitary gland, maintains the menstrual cycle. The menstrual cycle is now considered the fifth vital sign, reflecting an individual’s reproductive and general health status [37]. The uterine lining (i.e., endometrium) undergoes monthly successions of hormone-regulated endometrial tissue proliferation, preparation for embryo implantation (decidualization), shedding (menstruation), and tissue regeneration. Decidualization is the differentiation of endometrial stromal cells (eSCs) into decidual cells, which create a specialized niche for embryo implantation and placenta formation [38,39]. At the end of gestation, the uterus acts as a contractile organ for parturition. While most associate the term ‘uterus’ with pregnancy, among humans, its time spent in the ‘non-pregnant state’ far exceeds its time in the ‘pregnant state’.

The human uterus and female reproductive system are relatively distinct from those of most other species, including those used to model and study human disease. First, menstrual cycles and menstruation (the cyclic shedding of the endometrial lining) are restricted to a mere 3% of all animal species (including humans, some non-human primates (NHPs), several bats, the spiny mouse, and the elephant shrew) [40]. Humans and other higher primates have a simplex uterus with a single, undivided cavity lacking uterine horns [41,42]. By contrast, elephants, pigs, dogs, and cats have a Y-shaped bicornuate uterus with two distinct horns that house multiple fetuses during a single pregnancy [41,43]. Mice, rats, other rodents, marsupials, and rabbits have a duplex uterus, another type of uterus characterized by two separate and independent uterine horns that permit multiple fetuses in a single pregnancy [41,43]. Thus, the human simplex uterus is the least common among mammalian species and difficult to mimic in the lab.

### 3.2. Uterine Health

Numerous conditions affect the uterus, including endometrial cancer, endometriosis, adenomyosis, chronic endometritis, uterine fibroids, and PCOS. Except for endometrial cancer, these conditions are common. Also, these conditions can be associated with inflammation, chronic pain, abnormal menstrual bleeding, and infertility and, thus, negatively impact patients’ quality of life and reproductive potential.

Nevertheless, these uterine conditions remain underfunded and understudied [44,45,46,47,48]. Lack of research has led to significant diagnostic delays [49,50,51] and limited treatment options. Medical practitioners rely heavily on hormone-based therapies and, in some cases, surgical interventions (e.g., hysterectomy), which may have significant side effects and long-term health consequences [52,53]. There is a critical need for research aimed at better understanding uterine-related conditions to develop safer and more effective therapeutic options as well as preventative measures [54].

### 3.3. Multiple Approaches to Studying Human Uterine Conditions

Given the financial, ethical, and other challenges of conducting research with humans [55] and NHP [56], many investigators have relied on in vitro models using human uterus-derived cells and laboratory animals.

In vitro studies examining the effects of quercetin on uterine cells have mainly relied on primary human uterine cells and related cell lines (Table 1), mostly obtained via invasive endometrial biopsies and/or hysterectomies. Thus, access to primary control-eSCs and other endometrial cells from healthy individuals has been limited. A logistically less difficult and generally overlooked source of endometrial cells is menstrual effluent (or menstrual blood). Menstrual effluent contains shed non-immune cells (e.g., eSCs and epithelial) and immune cells (e.g., lymphocytes and myeloid) that can be easily and non-invasively collected each month [57]. Menstrual effluent provides endometrial cells from a diverse patient population, including healthy controls and various patient populations for pre-clinical, clinical, and population-based studies focused on uterine health [22,23,57,58,59,60,61].

Animal studies: Most pre-clinical animal models testing quercetin in uterine conditions have relied on common laboratory animals, namely, mice and rats (Table 2). Despite key differences between humans and common laboratory animals related to uterine health, numerous animal models have been developed to study human disease pathogenesis and develop therapies.

Human studies: Historically, women have been underrepresented in clinical trials. As indicated earlier, of the 120-plus clinical trials involving quercetin listed [1], few focus on women’s health, specifically uterine health. Not surprisingly, few clinical trials have investigated quercetin as a potential therapy for various uterine conditions (Table 3).

## 4. Therapeutic Effects of Quercetin Relevant to Uterine Conditions

**Figure 2:**
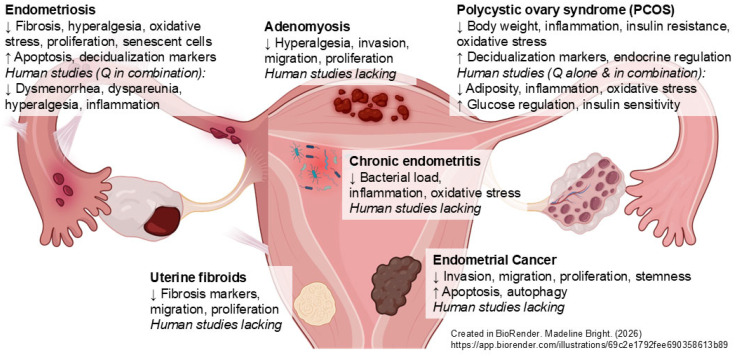
Pre-clinical and clinical evidence supporting the beneficial effects of quercetin on various uterine conditions (outside of pregnancy). ↓ = decrease; ↑ = increase. See Table 1, Table 2 and Table 3 for details.

As summarized above and shown in Figure 2, pre-clinical and clinical studies report quercetin’s diverse biological activities that may be beneficial for several aspects of uterine health.

### 4.1. Endometrial Cancer

Endometrial cancer is the sixth most common cancer in women globally [62], with the highest rate reported in high-income countries [63]. Several studies implicate imbalanced estrogen (E2) and progesterone (P4) signaling, obesity, nulliparity, PCOS, and Lynch syndrome as major risk factors [64]. Some data suggest that environmental exposures (e.g., endocrine disruptors) increase the risk of endometrial cancer [65]. While the diagnosis of uterine cancer cases has increased across all age groups, cases have doubled in women under 40 years of age [62]. One research priority is to screen and identify agents that may improve patient survivorship.

**In vitro studies:** Several in vitro models support the anti-proliferative effects of quercetin in the setting of endometrial cancer (Table 1). Specifically, quercetin (25–100 µM) significantly inhibited cell proliferation and reduced markers of cancer cell stemness using human endometrial carcinoma cell lines, EMN8 and EMN21 [66], possibly by suppressing STAT3 signaling. Similarly, quercetin (10–100 µM) significantly reduced the proliferation of Ishikawa (human endometrial adenocarcinoma) cells, with concomitant inhibition of growth-promoting *EGF* mRNA expression and cell cycle regulator cyclin D1 protein levels [18]. Using both Ishikawa and HEC-1A (human endometrial adenocarcinoma) cell lines, anti-proliferative, pro-apoptotic, anti-migratory, and anti-invasive activities of quercetin (100 µM) have been reported [67].

**Animal studies:** While rodent models of endometrial cancer have been developed [68,69], no published studies were found investigating quercetin’s potential therapeutic effects. However, quercetin’s role in doxorubicin-induced uterine toxicity has been explored [70] (Table 2). Doxorubicin (Adriamycin^®^) is a widely used chemotherapy administered to patients with solid tumors, soft tissue and bone sarcomas, and hematologic cancers [71]. Doxorubicin inhibits both DNA and RNA synthesis, blocking tumor cell proliferation; it promotes apoptosis and causes significant reactive oxygen species (ROS) production. Numerous adverse effects (e.g., gastrointestinal issues, bone marrow suppression, cardiotoxicity, nephrotoxicity, and reproductive toxicity) limit its use [71]. In a rat model of doxorubicin-induced uterine toxicity, quercetin (20 mg/kg/day) administered orally for 21 days significantly improved perimetrium and myometrium volume, as well as uterine gland and uterine vessel volumes when compared to doxorubicin alone [70].

**Human studies/human trials:** To our knowledge, there are no published reports of human studies examining quercetin as a potential treatment for endometrial or uterine cancer (note: over 4000 human clinical trials related to uterine or endometrial cancer are listed on clinicaltrials.gov). About 20 clinical studies investigated quercetin in other cancers; none tested quercetin in the setting of endometrial or uterine cancers [1].

### 4.2. Endometriosis

Endometriosis is characterized by the abnormal growth of uterine-like tissues outside the uterus (mainly in the abdominal cavity), affecting about 10% of reproductive-age women and teens [72,73,74]. Definitive diagnosis requires laparoscopy with histopathologic confirmation of stromal cells and glandular epithelial cells in lesion biopsies [75]. Symptoms include chronic pelvic pain, dysmenorrhea, dyspareunia, and dyschezia [72,76]. Patients experience hormonal imbalances (elevated E2 relative to P4) and P4 resistance (the inability of target tissue to respond to P4), which together promote inflammation and dysfunctional eSCs and impair fertility [72,77]. Approximately 30–50% of patients with endometriosis suffer from infertility [78]. Other challenges include delays in diagnosis, limited effective and tolerable treatments, and a lack of preventive therapies and cures [72,76].

Several studies have described alterations in the endometrium and specifically eSCs from patients with endometriosis, including aberrant inflammation and P4 resistance [79,80,81,82,83,84,85,86,87,88]. Also, recent single-cell RNA-sequencing of endometrial tissues (via menstrual effluent and endometrial biopsies) reveals numerous transcriptomic changes in eSCs and other endometrial cell types from endometriosis patients compared to healthy controls [89,90].

A key uterine abnormality implicated in endometriosis-related infertility is impaired decidualization [57,58,86,91,92]. Decidualization is the P4-driven differentiation of eSCs into nutritive and growth-supporting decidual cells required for human embryo implantation and, thus, pregnancy [38]. Decidualization can be modeled in vitro by treating human primary eSCs and related cell lines with P4 or P4 analogues (e.g., medroxyprogesterone acetate, MPA) with or without a cell-permeable form of cyclic adenosine monophosphate (cAMP) and then measuring decidualization biomarkers (e.g., insulin growth factor binding protein 1, IGFBP1, and prolactin, PRL) [38].

**In vitro studies:** As shown in Table 1, many studies support that quercetin (5–100 µM) enhances the decidualization response of primary human eSCs, including those obtained from endometriosis patients [20,22,23,93]. This pro-deciduogenic activity of quercetin is often accompanied by reduced eSC proliferation, likely through PI3K/AKT and ERK signaling pathways [20,22]. Thus, quercetin may limit the growth of endometriosis lesions and improve uterine decidualization responses and, hence, fertility. Consistent with these observations, quercetin (50 mg/kg) administered for three consecutive days every two weeks for ten weeks, combined with dasatinib, another senolytic agent, to young and old mice led to decreased activation of the PI3K/AKT1/mTOR signaling pathway in their uterine lining tissues and reduced signs of uterine aging and fibrosis [27]. Other studies support the senolytic activities of quercetin [22,23,93], raising the potential of targeting senescent cells and senescence-associated secretory phenotypes or SASPs (that promote inflammation) to treat endometriosis and other uterine conditions accompanied by increases in senescent and pro-fibrotic cells.

**Animal studies:** Animal models of endometriosis can be classified as induced (not naturally occurring) or spontaneous (i.e., develop naturally). One common feature among animals that naturally develop endometriosis is menstruation, which occurs in about 3% of species [40]. ‘Spontaneous’ models mainly employ NHPs, but because endometriosis affects only about 1 in 10 animals, investigators usually promote disease development by injecting endometrial tissues intraperitoneally (IP) [94,95]. These NHP models mimic the pathologic features of human endometriosis lesions [94] and have been helpful for better understanding disease initiation, progression, and endometriosis-associated infertility [95,96]; few studies have tested potential therapies [97]. Quercetin has not yet been tested in NHP models of endometriosis.

Despite their limited translatability, common laboratory animals (e.g., rats and mice) are routinely used to model endometriosis [98]. The two main types of rodent endometriosis models are autologous transplantation (placing endometrial/uterine tissue derived from genetically similar donor mice into recipient mice with an intact mouse immune system) and xenograft transplantation (placing human endometrial/uterine biopsy tissue into immunodeficient mice or mice transplanted with a human immune system). For most rodent transplant models, endometrial and/or uterine tissues are collected by mechanical separation/chopping or via punch biopsies of the donor uterus and then they are either injected IP into recipient animals or sutured onto the peritoneal lining of the recipient animals. To date, quercetin has been tested in rat and mouse autologous models of endometriosis (Table 2).

Using a rat implant model and quercetin treatment (375 mg/kg/day) for 21 days, Cao et al. observed reduced serum FSH and LH levels, reduced estrogen receptor alpha (ERα) and progesterone receptor (PGR) expression in the hypothalamus and eutopic and ectopic endometrial tissues, as well as increased estrogen receptor beta (ERβ) in the eutopic and ectopic endometrial tissues [99]. Lower quercetin doses were less effective. Quercetin likely acts as an anti-E2 and anti-P4 agent by reducing their binding to their receptors [99].

The anti-proliferative activity of quercetin was first demonstrated in a mouse model of endometriosis in 2019 [20]. In this study, quercetin (35 mg/kg) was injected IP every 3 days for 35 days, and it reduced implanted lesion size with concomitant reduction in *CCND1* mRNA expression (*CCND1* encodes cyclin D1, a critical cell cycle regulator) [20]. These anti-proliferative effects were replicated in a rat model where daily quercetin (15 mg/kg) for 30 days significantly reduced the size of implanted endometriosis lesions, while decreasing serum E2 and pro-inflammatory TNF levels and reducing markers of oxidative stress in the ectopic lesions [100]. Similarly, in a mouse model of endometriosis induced by the IP injection of endometrial tissues, daily quercetin (100 mg/kg × 14 days) significantly reduced endometriosis lesion size, purportedly by downregulating the expression of nuclear receptor 4A1 (NR4A1, implicated in fibrosis in ovarian endometriomas) [101].

**Human studies/human trials:** As shown in Table 3, a clinical study that included 33 women recently diagnosed with endometriosis reported that daily oral administration of 200 mg quercetin combined with 210 mg of curcuma longa (also known as turmeric) and 150 mg of N-acetylcysteine for 2 months significantly reduced dysmenorrhea, dyspareunia, and pelvic pain [102], characteristic features of endometriosis. Treatment was also associated with decreased use and lower doses of non-steroidal anti-inflammatory drugs. In addition, no significant adverse effects of the combined treatment were reported, suggesting quercetin combined with curcuma longa and N-acetylcysteine could be a well-tolerated anti-inflammatory adjuvant treatment for endometriosis [102]. The contribution of quercetin to these outcomes is unknown.

Another clinical study recruited 90 patients with endometriosis; one third were treated with a placebo, one third were treated with linseed oil and 5-methyltetrahydrofolate, and one third were treated with a dietary supplement that included quercetin (200 mg) along with omega-3 and omega-6 fatty acids, nicotinamide (20 mg), 5-methyltetrahydrofolate (400 µg), titrated turmeric (20 mg), and titrated parthenium (19.5 mg) [103]. All participants were asked to consume a special diet with increased fiber and omega-3 fatty acids, as well as reduced dairy, meat, gluten, caffeine, alcohol, chocolate, saturated fat, butter, and margarine, and free of soy, aloe, and oats throughout the three-month study. Using the Visual Analog Scale for pain and related symptoms, the 30-participant group (mean age 35.2 years) treated with the quercetin supplement reported significant reductions in headaches (14% to 4%), cystitis (12% to 2%), muscle aches (4% to 1%), irritable colon (15% to 6%), dysmenorrhea (62% to 18%), dyspareunia (30% to 15%), and chronic pelvic pain (62% to 18%) when compared to the placebo group, as well as no adverse effects.

### 4.3. Adenomyosis

Adenomyosis (also called ‘endometriosis of the uterus’) is characterized by deep invasion of endometrial glandular epithelial cells and stromal cells into the myometrium (either diffusely or focally), leading to chronic pelvic pain, excess uterine bleeding (heavy periods), and infertility [104]. Risk factors include a history of uterine surgical procedures (e.g., C-section), increasing age, and parity [105]. Although adenomyosis shares some features with endometriosis, including lesions consisting of endometrial-like tissues [77], in adenomyosis, the endometrial cells invade the muscular outer layer of the uterus (known as the myometrium) [106], whereas endometriosis lesions implant outside of the uterus. The overall incidence rate of adenomyosis in the US is estimated to be 1%, with a higher incidence among those of African ancestry [107]. However, due to diagnostic challenges, the true incidence and prevalence of adenomyosis are not known. Patients experience high rates of infertility, and more than 80% of patients undergo hysterectomies, imposing significant emotional, social, physical, and economic burdens [50,104,105,108]. There is high use of chronic pain medications among patients with adenomyosis [104], as there are limited treatment options. Thus, one important goal is to identify treatments that reduce adenomyosis symptoms and progression and maintain fertility.

**In vitro studies:** Primary eutopic eSCs and ectopic eSCs (isolated from uterine tissues and adenomyosis lesions collected from patients with adenomyosis, respectively) were examined for alterations in proliferation, migration, and invasive activity following treatment with quercetin (25–80 µM and 20–160 µM, respectively) [21] (Table 1). Quercetin significantly reduced the proliferation of ectopic adenomyosis eSCs and inhibited cell mobility and invasive potential of both eutopic and ectopic adenomyosis eSCs, with greater effects on ectopic adenomyosis eSCs compared to vehicle-treated cells [21]. These inhibitory effects were associated with reduced expression of proteins implicated in cell migration and invasion (ezrin, fascin, MMP-2, and MMP-9) [21]. MMP-2 and MMP-9 degrade the extracellular matrix (ECM) to aid cell migration and invasion and have been targeted for treating various cancers [109,110], albeit with limited success.

**Animal studies:** Adenomyosis is not observed in laboratory animals and must be induced by mechanical damage, pituitary engraftment, human tissue xenotransplantation, or neonatal tamoxifen administration [111]. One study examined quercetin’s possible therapeutic effect using the tamoxifen-induced mouse model [112] (Table 2). Quercetin (25–50 mg/kg/day, orally) for 21 days significantly reduced the depth of endometrial cell infiltration into the myometrium [112]. Quercetin treatment also significantly reduced adenomyosis-associated hyperalgesia, possibly by reducing levels of TRPV1 (Transient Receptor Potential Vanilloid 1). TRPV1 is implicated in detecting pain sensation and regulating neural pathways associated with central pain sensitization. This reduction in pain is consistent with several studies describing quercetin’s analgesic and anti-nociceptive effects mediated by suppressing inflammation and oxidative stress, as well as modulating GABAergic and opioidergic systems [113,114,115].

**Human studies/human trials:** To our knowledge, there are no published reports of human studies examining quercetin as a potential treatment for adenomyosis. Among the 127 clinical trials listed on clinicaltrials.gov investigating adenomyosis, none report testing quercetin as a possible intervention [1].

### 4.4. Chronic Endometritis/Uterine Infections

Chronic endometritis is clinically defined as persistent endometrial inflammation, often of microbial origin [116,117,118]. The most common bacteria implicated are *Escherichia coli*, *Enterococcus faecalis*, and *Streptococcus* species, *Staphylococcus* species, as well as mycoplasma and *Ureaplasma urealyticum* [117,118]. While the true prevalence of chronic endometritis is not known, it is believed to contribute to infertility, pregnancy losses, and poor pregnancy outcomes [119,120]. Interestingly, chronic endometritis is considered a risk factor for endometriosis [121,122]. Among patients with unexplained infertility, chronic endometritis is diagnosed in about 10–60% of patients, depending on the study [118,123,124,125,126,127]. Unfortunately, misdiagnosis is common [128,129]. Accurate definitive diagnosis is challenging because it requires an endometrial biopsy followed by assessment of plasma cells within an area of dense endometrial stroma, without clear guidelines for the number of plasma cells required for a positive diagnosis [116,126,127]. Because diagnosis relies on an invasive procedure not routinely performed, and patients often lack specific symptoms, chronic endometritis is rarely identified outside the setting of infertility (a major symptom). Although antibiotics are relatively effective in reducing infection [128,129], about 20–25% of cases may stem from non-infectious causes (e.g., intrauterine devices or IUDs, polyps, and uterine anomalies) [129].

**In vitro studies:** The most common model of chronic endometritis employs human eSCs or other endometrial cells treated with lipopolysaccharide (LPS), the main pathogen-associated molecular pattern or PAMP released by Gram-negative bacteria. However, numerous bacteria, including Gram-positive, Gram-negative, mixed, and Gram-variable bacteria, as well as those lacking a bacterial cell wall (e.g., mycoplasma), are implicated in chronic endometritis [127]. Additionally, this model does not accurately mimic non-infectious forms of chronic endometritis. While numerous studies have reported the adverse effects of PAMPs on cultured endometrial cells, none have examined the effects of quercetin in this context.

**Animal studies:** Several studies support that quercetin-containing plant extracts exert anti-inflammatory and antioxidant effects in animal models of chronic endometritis (Table 2). Using a murine model of LPS-induced chronic endometritis, oral administration of total flavonoids from *Clinopodium chinense*, a mint plant with high total flavonoid content that includes quercetin as a major component, at 100–400 mg/kg/day starting one day after LPS treatment and continuing for 7 days significantly reduced LPS-induced uterine oxidative stress and inflammation [130]. Specifically, treatment with total flavonoids of *Clinopodium chinense* (*TFC*) significantly decreased myeloperoxidase (MPO) activity, pro-inflammatory cytokines (IL-1β, IL-18, and TNF), and activation of the NLRP3 inflammasome in endometrial tissues exposed to LPS compared to control tissues exposed to LPS [130]. These data support the strong anti-inflammatory and antioxidant activities of TFC (containing quercetin as a major component) in the endometrium. Interestingly, *Clinopodium chinense* has been used to treat uterine bleeding and other hemorrhagic conditions for centuries [131].

Another study using a mouse model of LPS-induced endometritis evaluated Tiaoqi Jiedu formula, a ‘de-toxifying’ herbal mixture that contains quercetin as a main component [132]. The authors described that low, medium, and high doses of quercetin-containing Tiaoqi Jiedu formula reduced uterine tissue inflammation and systemic pro-inflammatory cytokines (TNF, IL-6, IL-1β, IL-8) and increased IL-10 levels, a potent anti-inflammatory mediator [132]. Treatment with the quercetin-containing herbal mixture decreased uterine pyroptosis markers (NLRP3, GSDMD, and caspase-1) implicated in a form of inflammatory cell death and reduced TLR4 activation, which, in turn, blunted NF-κB-mediated inflammation. This was a ‘prophylaxis study’ where mice were treated with the Tiaoqi Jiedu formula orally twice daily for 28 days prior to LPS injection into the uterus, and the uterine tissues were analyzed 24 h post-LPS injection.

An additional study examined the effects of a quercetin-containing plant extract on antioxidant, anti-inflammatory, and antimicrobial properties after establishing bacteria-induced endometritis in rats [133]. In this study, adult female Wistar rats with Gram-positive *Staphylococcus aureus*-induced bacterial endometritis or Gram-negative *Escherichia coli*-induced bacterial endometritis were treated with an extract made from leaves of the *Eucalyptus robusta* plant (25 mg/kg/day, orally for 5 days) or cefixime, a third-generation cephalosporin broad-spectrum antibiotic (15 mg/kg/day by oral gavage for 5 days) [133]. Treatment with either the quercetin-containing *Eucalyptus robusta* extract or the antibiotic for 5 days significantly reduced bacterial load and showed significant antioxidant (reduced myeloperoxidase (MPO), inducible nitric oxide synthase (iNOS), and nitric oxide (NO) levels), anti-inflammatory (reduced expression of TLR4, TLR9, and IL-10), and uterine tissue protective effects [133]. Although quercetin is a main component in these plant extract studies, it is unclear to what extent quercetin mediated the beneficial effects.

**Human studies/human trials:** No published human studies examining quercetin as a treatment for human chronic endometritis were found. Among the 21 clinical trials listed on clinicaltrials.gov related to chronic endometritis, none tested quercetin as a possible therapy [1].

### 4.5. Uterine Fibroids (Or Leiomyomas)

Uterine fibroids affect more than 75% of women, and about 25% of these patients experience significant fibroid symptoms (e.g., pelvic pressure and pain, abnormal, prolonged, or heavy menstrual bleeding (with iron-deficient anemia in severe cases), and infertility) [134,135]. In addition to bothersome heavy menstrual bleeding and lost days of work and school, uterine fibroids contribute to more than 30% of all hysterectomies, outpacing hysterectomies related to endometrial cancer [134]. The social and economic burdens of hysterectomies are significant, leading to loss of fertility and libido, early menopause symptoms, depression, reduced bone health, and increased cardiovascular disease risks [134,135]. Additionally, women of African ancestry disproportionately suffer from uterine fibroids; they experience larger and more fibroids, are affected at younger ages, and have hysterectomies at younger ages than White women [134,135]. While the exact causes of uterine fibroids are not fully understood, age, genetics, ancestral background, endogenous and exogenous hormonal factors, obesity, and stress are implicated in promoting the transformation of normal myocytes into abnormal myocytes with unrestricted growth potential [135,136]. Although they lack metastatic potential, uterine fibroids are comprised of fibrous tissues rich in ECM that can contribute to pathologic fibrosis [137]. Few treatments are available, including hormonal contraceptives, GnRH agonists, and surgery (myomectomy and hysterectomy); surgery remains a common intervention [138].

**In vitro studies:** Consistent with prior studies supporting quercetin’s anti-fibrosis activities in animal models of hepatic and pulmonary fibrosis [139,140,141], quercetin (at >30 µM) has been reported to exert anti-fibrotic effects using primary cells isolated from human myometrium and uterine fibroids [142]. Greco and co-workers demonstrated that ex vivo quercetin treatment of both human myometrial and uterine fibroid cells significantly decreased expression of pro-fibrotic *Col1A1* and *FN* mRNA (which encode collagen 1A1 and fibronectin, respectively) as well as fibronectin protein production and significantly inhibited cell proliferation and migration [142]. The expression of ECM proteins, collagen 1A1 and fibronectin, is dysregulated in uterine fibroids, contributing to increased tissue stiffness, fibrosis, tissue growth, and disease progression and, thus, represents potential therapeutic targets of quercetin.

**Animal studies:** Animal models of uterine fibroids include spontaneous, genetically modified, hormone-induced, and xenograft approaches [143]. No studies that have examined the effect of quercetin on uterine fibroids using animal models were found.

**Human studies/human trials:** No published reports of human studies exploring the use of quercetin for treating uterine fibroids were found. Almost 500 clinical trials for uterine fibroids are listed on clinicaltrials.gov; none included quercetin as a potential treatment [1]. However, there is one study examining uterine fibroids for the presence of senescent cells (NCT06135870) and several fibrosis-related trials employing quercetin (with or without dasatinib) either in vivo or ex vivo using patient-derived cells (NCT02874989, NCT05506488, NCT00512967) [1].

### 4.6. PCOS (Polycystic Ovary Syndrome)

PCOS is characterized by hormone dysregulation and insulin resistance accompanied by irregular menstrual cycles with ovulatory dysfunction, excess androgen production, ovarian cysts, and infertility [144]. In 2019, the prevalence of PCOS was 5.2% in the United States [145]. As a common condition, PCOS poses enormous economic burdens related to treating and managing metabolic and hormonal dysfunction, infertility, depression, and anxiety [146]. Its pathogenesis is mediated through complex interactions between genetics, environment, and hormone imbalances, with inflammation and insulin resistance promoting androgen overproduction by the ovaries, leading to improper follicle maturation and irregular menstrual cycles, as well as ovarian cysts and metabolic syndrome/metabolic and endocrine abnormalities [147,148,149]. The heterogeneity of PCOS among patients is a major challenge when using various models to study it.

There are limited treatment options for PCOS, namely, metformin and drugs that target hormone dysregulation (e.g., oral contraceptives, anti-androgens) [144]. No treatments for PCOS are FDA-approved. New therapies, including kisspeptin-based treatments, neurokinin 3 receptor antagonists, and glucagon-like peptide 1 (GLP-1) agonists, show promising results in clinical trials [144]. Several studies describe PCOS-related uterine alterations, and based on quercetin’s metabolic and anti-inflammatory effects, it is not surprising that quercetin supplementation has shown benefit for some women with PCOS [10,12,33,150].

**In vitro studies:** A recent single-nuclei RNA-sequencing study describes PCOS-specific alterations in various endometrial cells, including markers associated with reduced endometrial receptivity and decidualization [151]. These findings reflect previous reports that PCOS-derived eSCs have decidualization defects [152,153,154,155]. While no studies have directly examined the effect of quercetin on eSC decidualization using primary human PCOS-derived eSCs in vitro, Wang et al. used the T-HESC cell line (hTERT-immortalized fibroblast-like uterine cells from a patient with non-malignant myomas) to show that insulin (50 nM, to mimic the compensatory hyperinsulinemia due to insulin resistance in the setting of PCOS) inhibited decidualization, as measured by reduced *IGFBP1* and *PRL* mRNA expression, as well as *IRS1/2* mRNA expression [150]. Furthermore, quercetin (10 µM) reversed this inhibitory effect and increased *IRS1/2*, *GLUT2*, and *GLUT4* mRNA expression [150] in T-HESCs.

**Animal Studies:** PCOS has been widely modeled in rodents using LTZ (letrozole), DHEA (dehydroepiandrosterone), and TP (testosterone propionate), with or without high-fat diets [156]. DHEA is the first androgen circulating in female adolescents, and levels are elevated in about 25% of patients with PCOS [157]. LTZ is an aromatase inhibitor that blocks the conversion of testosterone (T) to E2, leading to hyperandrogenism [158]. TP directly promotes hyperandrogenism. Typically, pre-pubertal rodents are injected daily with DHEA [159], LTZ [160], or TP [161] for several weeks, leading to altered endocrine function (increased T and LH), increased ovarian weight, anovulation, ovarian cysts, and atretic follicles, as well as disrupted estrous cyclicity in adult animals [156]. While most PCOS studies focus on the ovaries, persistent endocrine and metabolic alterations [149] and chronic inflammation [148] associated with PCOS are implicated in endometrial changes, including abnormal estrous cycles and infertility.

Numerous studies investigating quercetin and quercetin-containing treatments in animal models of PCOS reveal that quercetin or quercetin-containing extracts improve PCOS-related features (e.g., metabolic, endocrine, hormone, inflammation, etc.) (Table 2). For example, the effect of Yishen Huatan and Huoxue decoction (YHHD), a traditional Chinese medicine formula containing quercetin, has been studied using a DHEA-induced mouse model of PCOS [150]. Treatment of PCOS-mice with YHHD improved T and LH levels and glucose metabolism [150]. At the level of the endometrium, YHHD treatment improved decidualization and estrus cyclicity [150]. Using the same model, purified quercetin (50 mg/kg, subcutaneously) administered daily for 20 days improved decidualization, T and LH levels, and glucose metabolism [150]. Consistent with these findings, patients with PCOS treated with YHHD had lower early miscarriage rates compared to those not treated [150].

Purified quercetin improves P4 levels and normalizes the estrous cycle in several animal models of PCOS (Table 2). Specifically, using an LTZ-induced PCOS rat model, oral quercetin (30 mg/kg) administered daily for 21 days reduced androgen levels and CYP17A1 expression (a key enzyme involved in androgen synthesis), increased P4 (a key regulator of endometrial function), and decreased the E2/P4 ratio, which together restore estrous cyclicity [162]. Similarly, oral administration of quercetin at 100 mg/kg daily for 15 days significantly improved P4 levels and estrous cycling and decreased pro-inflammatory cytokine levels in a rat model of DHEA-induced PCOS [163]. Other studies using rodent models of PCOS report that quercetin reduces inflammatory mediator production and oxidative stress and promotes anti-inflammatory mediator production [163,164,165]. These anti-inflammatory effects may have important consequences for the endometrium.

Quercetin (15 mg/kg/day) provided orally for 30 days to rats following DHEA-induced PCOS increased adiponectin, adiponectin receptor 1, and nesfatin expression in the uterus [166]. Both adiponectin (via the adiponectin receptor) and nesfatin exert anti-inflammatory effects [167,168] and regulate pathways related to metabolic syndrome [166,169,170]. Also, oral quercetin (100 mg/kg daily for 30 days) following the induction of PCOS by LTZ in rats significantly increased adiponectin levels [32]. Coincidentally, adiponectin expression in the human endometrium is highest during the ‘window of implantation’ in the mid-secretory phase, and loss of endometrial adiponectin receptor expression has been reported in patients with recurrent implantation failure vs. fertile women [171].

Ovarian and endocrine changes, chronic inflammation, and metabolic alterations observed in PCOS impact the uterus and its function. For example, endometrial P4 resistance (defined as decreased responsiveness of target endometrial tissues to bioavailable P4) occurs with PCOS. P4 resistance in endometriosis and PCOS is linked to infertility [172,173]. While the cause of P4 resistance in PCOS and other patients is unclear, there is supporting evidence that chronic inflammation contributes [88,174,175]. Similarly, the cause of metabolic dysfunction in the setting of PCOS is not well understood.

**Human studies/human trials:** Quercetin supplementation in women with PCOS exerts anti-inflammatory and endocrine effects, including improved insulin sensitivity [33], decreased T levels [10,12,33], and decreased LH levels [10,12,33], with minimal adverse effects (Table 3).

In a randomized, double-blinded study, 31 women with PCOS, aged 20–37 years, were treated with quercetin (500 mg/day) for 40 days, vs. 33 women with PCOS, aged 20–37 years, treated with placebo tablets [13] (Table 3). The participants receiving quercetin showed significant decreases in circulating LH, TNF, and IL-6 levels compared to pretest levels, while participants receiving the placebo showed no change from their pretest values.

Another double-blind and placebo-controlled trial included 78 overweight or obese women with PCOS who received either quercetin (1000 mg/day) or placebo for 12 weeks [10] (Table 3). Metabolic, hormonal, and inflammatory markers were assessed before and after treatment. Quercetin supplementation significantly reduced plasma resistin levels, as well as serum T and LH levels, when compared with the placebo group. Elevated resistin levels in PCOS patients are proposed to drive inflammation, metabolic dysfunction, and hyperandrogenism [176]. A recent meta-analysis of the effects of quercetin treatment for PCOS provides similar support [177]. Although fasting glucose, insulin, and insulin resistance indices improved in the quercetin group, these changes were not significantly different from the placebo group, indicating that quercetin’s primary effects may be on resistin-related inflammatory pathways and androgen regulation rather than glucose homeostasis [176]. Although little is known about how resistin affects the endometrium, resistin binds to functional receptors expressed in the endometrium, including an isoform of decorin, a stromal proteoglycan [178], and the LPS receptor TLR4 [179]. Decorin is induced by P4 and mediates the inhibition of stromal cell proliferation [180]. The binding of resistin to TLR4 mediates pro-inflammatory effects [181]. Thus, quercetin may act through multiple different pathways.

Overall, these findings and others suggest that oral quercetin supplementation may mediate potent anti-inflammatory and beneficial endocrine effects on the endometrium in patients with PCOS and support future larger validation studies.

**Table 1:** **In vitro studies that examine the effects of quercetin (Q) on endometrial cells.**

Q, Source (If Given)	Q Dose(s)	Cells: Primary (1°) or Cell Line (CL)	Q Functions	Specific Outcomes	Reference
**Adenomyosis**					
Q, Sigma Aldrich CAS 6151-25-3	25–80 μM (Eu); 20–160 μM (EE)	1°: HeSCs (Eu, EcE)	AM, AP	↓ migration, proliferation; ↓ invasion	[21]
**Endometrial Cancer**					
Q, China	25–100 μM	CL: EMN8, EMN21 (endometrial carcinoma)	AP	↓ proliferation; ↓ stemness; ↓ sphere formation; ↓ ERα; ↓ STAT3/JAK2 signaling;	[66]
Q, N/A	10–100 μM	CL: Ishikawa (endometrial adenocarcinoma)	AP	↓ proliferation; ↓ EGF & cyclin D1; ↑ VEGF	[18]
Q, N/A	100 μM	CL: Ishikawa and HEC-1 A cells (endometrial adenocarcinoma)	AM, AP, PA	↓ proliferation; migration & invasion; ↑ apoptosis & autophagy	[67]
**Endometriosis**					
Q, Sigma Aldrich, USA	5–20 μM	1°: HeSCs (Eu); CL: epithelial cells (VK2/E6E7, vaginal); CL: (End1/E6E7, endocervix)	AP, AO	↓ proliferation, cyclin D1; ↓ ROS; ↓ phosphorylation ERK1/2, P38, AKT, P70S6K and S6 proteins;	[20]
Q, Tokyo Chemical Industry	20–100 μM	1°: HeSCs (Eu)	AP, PD, S	↑ decidualization; ↓ senescent markers; ↓ *TP53*	[93]
Q, Sigma Aldrich, USA	25–100 μM	1°: HeSCs (Eu)	AF	↓ TGFβ-induced fibrotic changes in HeSCs (↓ COL1A1, α-SMA, FN)	[182]
Q, Indofine, USA	50–150 µM	1°: HeSCs (Eu); IHeSCs (Ec); IHeSCs; CL: Ishikawa cells	AP	↓ proliferation; ↓ pro-fibrosis markers	[101]
Q, Fisher, USA CAS 849061-97-8	6–50 μM	1°: ME-eSCs (Eu)	AI, AP, PA, PD, S	↑ decidualization; ↓ senescent markers; ↓ migration; ↑ phosphorylation AKT, ERK1/2, PRAS40, p53; ↑ total p53; ↑ apoptosis;	[22,23]
**Uterine Fibroids/Leiomyomas**					
Q, Sigma-Aldrich, Milan, Italy	33–827 µM	1°: MYO & UF cells	AF, AM, AP	↓ Col1A1 and FN; ↓ migration & proliferation for MYO cells (not UF cells)	[142]
**Polycystic Ovary Syndrome (PCOS)**					
Q, N/A	5–200 µM	CL: T-HeSCs	EE, PD, PA	↑ decidualization; ↑ GLUT2, GLUT4; ↓ IRS1/2	[150]

CAS: Chemical Abstract Service; CL: cell line; Ec: ectopic; EcE: ectopic endometrial cells in uterus (adenomyosis); ER: estrogen receptor; Eu: eutopic; HeSCs: human endometrial stromal cells; IHeSCs: immortalized HeSCs; ME-eSCs: menstrual effluent-derived endometrial stromal cells; MYO: myometrial; N/A: not available; Q: quercetin; ROS: reactive oxygen species; T-HeSCs: transformed HeSCs; UF: uterine fibroblasts. Functions: AF: anti-fibrotic; AI: anti-inflammatory; AM: anti-migratory; AP: anti-proliferative; AO: antioxidant; EE: endocrine effects; PA: pro-apoptotic; PD: pro-deciduogenic; S: senolytic. ↓ = decrease; ↑ = increase.

**Table 2:** **Animal studies that examine the effects of quercetin (Q) on uterine condition outcomes.**

Species (Model)	Q Source, Formulation & Combination	Q Doses & Duration	Route of Delivery	Q Functions	Specific Outcomes	Reference
**Adenomyosis**						
Mice (tamoxifen- induced)	Q Sigma-Aldrich CAS 117-39-5	25–50 mg/kg/d ×21 d	Oral	-	↓ hyperalgesia	[112]
**Endometritis**						
Mice (LPS- induced)	Total flavonoids from *C. chinense* (TFC with Q)	100–400 mg/kg/d ×7 d	Oral	AI, AO	↓ MPO (ROS); ↓ cytokines/inflammation; ↓ NLRP3	[130]
Mice (LPS- induced)	Tiaoqi Jiedu formula (contains Q)	N/A	N/A	AI	↓ uterine pathology/injury; ↓ serum TNF, IL-6, IL-1β, & IL-8; ↓ pyroptosis markers; ↓ TLR4→NF-κB signaling; ↑ IL-10	[132]
Rats (*Staph* *aureus* ± *E. coli*-induced)	Extract of *Eucalyptus Robusta* plant leaves (contains Q)	25 mg/kg/d ×5 d	Oral	AB, AI	↓ serum IL-1β & TNF; ↑ serum IL-10; ↓ bacterial load; ↓ uterine inflammation	[133]
**Polycystic ovary syndrome (PCOS)**						
Mice (DHEA- induced)	YHHD (contains Q)	N/A	N/A	EE	↓ body weight; ↓ FSH, LH, T; ↓ IR; ↑ decidualization	[150]
Mice (DHEA- induced)	Q, Aladdin Biochemical Technology Co., China	50 mg/kg/d ×20 d	Subcutaneous	EE, PD	↓ body weight; ↓ LH, T; ↓ IR ↑ decidualization	[150]
Rats (DHEA- induced)	Q, N/A	15 mg/kg/d ×30 d	Oral	AI, EE	↑ adiponectin, adiponectin receptor 1, nesfatin-1; ↓ aromatase, E2	[166]
Rats (LTZ-induced)	Q, N/A	100 mg/kg/d ×30 d	Oral	EE	↓ TGs, cholesterol, T, glucose; ↓ IR; ↑ E2, P4, adiponectin	[32]
Rats (LTZ-induced)	Q, N/A	30 mg/kg/d ×21 d	Oral	AI, AO, EE	↓ T and lipid peroxidation; ↓ cholesterol, TGs, LDL normalized E2/P4	[162]
Rats (DHEA-induced)	Q, Sigma in 1% sodium carboxy-methyl-cellulose	100 mg/kg 4×/d ×28 d	Oral	AI, AO	↓ NADPH oxidase activity; ox-LDL; fasting insulin, IR; TLR-4/OX-LDL/NADPH oxidase subunit p22phox; ↓ serum IL-1β, IL-6, TNF; OX-LDL->TLR-4->NFκB signaling	[165]
Rats (DHEA-induced)	Q, Sigma-Aldrich ≥95% (HPLC grade) in saline	25 mg/kg/d ×28 d	Oral	AF, AI, AO, EE	↓ Free T ↓ LH; ↓ LH/FSH Ratio ↑ E2; E2/Free T ↑ primordial, 1° & 2° follicles; eliminate cystic follicles; restored estrous cycle; ↓ apoptosis markers; ↑ cell survival markers improved metabolic outcomes	[31]
Rats (TP-induced)	Q, pure, Sigma	150 mg/kg 4×/d ×42 d	Oral	AI, AO, EE	↓ fasting insulin; ↓ T; ↓ serum cholesterol, TGs; improved uterine pathology; restored estrus cycle	[183]
**Endometriosis**						
Rats (implant model)	Q (98% pure) Swiss ALEXIS Biochemical Corp.	60–375 mg/kg/d ×21 d	N/A	EE	↓ LH, FSH; ERα, ERβ, PGR in hypothalamus, pituitary, and endometrium	[99]
Rats (implant model)	Q, Sigma, Germany	15 mg/kg/d ×30 d	N/A	AP, AO, EE	↓ lesion size; ↓ serum E2, TNF; ↓ oxidative stress & autophagy	[100]
Mice (endometrial tissue, IP)	Q, Indofine, USA	100 mg/kg/d ×14 d	IP	AP	↓ lesion size	[101]
Mice (implant model)	Q, Sigma Aldrich, USA	35 mg/kg every 3 d × 30 d	IP	AP	↓ lesion size	[20]
**Other: chemotherapy-induced uterine toxicity**						
Rats (DOX-induced)	Q, Sigma-Aldrich, in corn oil	20 mg/kg/d ×21 d	Oral	AO	↑ uterine volume & thickness; partial protection of ovary and uterus	[70]

d: day; DHEA: dehydroepiandrosterone; DOX: doxorubicin; E2: estrogen; FSH: follicle-stimulating hormone; IP: intraperitoneal; IR: insulin resistance; LH: luteinizing hormone; LPS: lipopolysaccharide; LTZ: letrozole; MPO: myeloperoxidase; N/A: not available; P4: progesterone; Q: quercetin; ROS: reactive oxygen species; T: testosterone; TFC: total flavonoids from *C. chinense*; TGs: triglycerides; TP: testosterone propionate; YHHD: Yishen Huatan Huoxue decoction. Functions: AB: anti-bacterial; AI: anti-inflammatory; AP: anti-proliferative; AO: antioxidant; EE: endocrine effects; PD: pro-deciduogenic. ↓ = decrease; ↑ = increase.

**Table 3:** **Human studies that examine the effects of quercetin (Q) on uterine-related outcomes.**

Subject/ Condition	Age Range (Years, yr); Sample Size	Q Formulation or Combination	Q Source	Q Dose & Duration (Delivery)	Q Functions	Specific Outcomes	Reference
**Endometriosis**							
Patients with stage IV Endo	Mean age = 34 yr; *n* = 90 (30 per group)	Supplement containing: 200 mg Q, fatty acids, 20 mg nicotinamide, 400 µg 5-methyltetrahydro-folate calcium salt, 20 mg titrated turmeric, 19.5 mg titrated parthenium (2×/d)	N/A	Q: 400 mg/d in supplement for 3 months with strict diet (oral)	AI	↓ PGE2 (AI); ↓ pain	[103]
Patients with Endo diagnosed for ≥3 months	>18–50 yr; *n* = 33	Supplement (allieNDO) containing: Q (200 mg) + curcuminoids + 150 mg NAC	N/A	1 tablet allieNDO/d with Q: 200 mg/d × 3 months (oral)	AI	↓ dysmenorrhea; ↓ dyspareunia, ↓ pelvic pain; ↓ NSAIDs	[102]
**Polycystic ovarian syndrome (PCOS)**							
Patients with PCOS	20–37 yr; *n* = 72	Q, N/A	N/A	Q: 500 mg/d ×40 d (oral)	AI, EE	↓ inflammation; improved hormones	[13]
Patients with PCOS	Median age ~30 yr; *n* = 660 (220 per group)	3 groups: dydrogesterone, Q-containing-YHHD, dydrogesterone + Q-containing-YHHD	N/A	Q dose, N/A 14–20 d (oral)		Q-containing YHHD + dydrogesterone group only: ↓ T; ↓ miscarriage rate (<20 wk gestation)	[150]
Patients with PCOS + obesity [BMI: 25–40 kg/m^2^]	20–40 yr; *n* = 78	Q, 500 mg capsules	Jarrow, USA	Q: 2.5 g/d ×12 wk (oral)	AA, AI, AO	↓ resistin, total T, & LH	[10]
Patients with PCOS + obesity [BMI 25–40 kg/m^2^]	20–40 yr; *n* = 84 (42 per group)	Q, 500 mg capsules	Jarrow, USA	Q: 1 g/d ×12 wk (oral)	AI, AO, EE	↑ adiponectin activity; ↓ IR, T, LH, fasting insulin, fasting blood sugar	[33]

BMI: body mass index; d: day; Endo: endometriosis; IR: insulin resistance; LH: luteinizing hormone; NAC: N-acetylcysteine; NSAIDs: non-steroid anti-inflammatory drugs; PCOS: polycystic ovary syndrome; PGE2: prostaglandin E2; Q: quercetin; T: testosterone; wk: weeks; YHHD: Yishen Huatan Huoxue decoction; yr: years. Functions: AA: anti-adipogenic; AI: anti-inflammatory; AO: antioxidant; EE: endocrine effects. ↓ = decrease; ↑ = increase.

## 5. Discussion: Limitations, Barriers, and Future Directions

Quercetin exerts numerous functions, including anti-inflammatory, anti-proliferative, antioxidant, anti-fibrotic, antimicrobial, and senolytic activities, as well as metabolic and endocrine-modulating properties [2] (Table 1, Table 2 and Table 3, Figure 2). Many of these functions are dysregulated in various uterine conditions highlighted in this review (Figure 2). Quercetin directly scavenges ROS to exert antioxidant effects and binds various proteins such as kinases, transporters, and transcription factors to regulate several signaling pathways, including those that overlap [3,184,185]. In the setting of cancer, quercetin exerts anti-tumor effects through the regulation of angiogenic activity (by reducing VEGF or vascular endothelial growth factor expression), cell proliferation (by regulating the expression of EGFR (epidermal growth factor receptor) and other cell cycle mediators and MAPK/ERK1/2 signaling), inflammation (via STAT3 and NFκB pathways), and cell survival and apoptosis (via PI3K/AKT and MAPK/ERK1/2 signaling and the expression of p53, an important tumor suppressor) [3]. In non-cancer conditions, quercetin regulates aberrant cell proliferation and survival by targeting the p53, p21, p27, cyclins, CDKs and Wnt/β-catenin, MAPK/ERK1/2 and PI3K/AKT signaling pathways; quercetin inhibits inflammation (via NFκB translocation and signaling) [184,185]. Quercetin’s ability to bind various target proteins implicated in multiple signaling pathways to exert cumulative and varying effects may explain how quercetin’s biological activities may improve multiple uterine condition-specific outcomes. Quercetin’s antimicrobial effects and senolytic activities may target conditions mediated by infections and senescent cells, respectively. Additional research is needed to better define the mechanisms underlying quercetin’s potential therapeutic effects within the uterus and reproductive tract because (1) signaling data is based on limited studies and under specific conditions (mainly in vitro studies using mono-cultures, which may not reflect in vivo findings); (2) signaling pathways are often analyzed at one point in time and signaling is a dynamic process; (3) many publications focus on a single pathway, excluding analysis of other signaling pathways; (4) cross-talk between signaling pathways occurs; and (5) signaling can be cell-specific, tissue/organ-specific, and context-specific (e.g., in the setting of cancer vs. non-cancer conditions).

In addition to the lack of understanding of quercetin’s therapeutic mechanisms, several limitations discussed herein temper the therapeutic potential of quercetin for treating uterine conditions. The translatability of both in vitro and animal studies is limited. It is difficult to translate the concentrations and doses of quercetin used in in vitro studies of animals to human studies. Similarly, the translational relevance of quercetin doses for animals vs. humans is unclear. As shown in Table 2, animals consumed 15–375 mg/kg per day for up to 6 weeks, while humans consumed 200–2500 mg/day for up to 3 months (Table 3) or 1.2 mg/kg/day–14 mg/kg/day for adult women and 1–12.5 mg/kg/day for adult men (based on typical body weight ranges for women and men) [186]. Multicenter randomized controlled trials will be required to confirm optimal quercetin doses and durations for each uterine condition. As discussed, the human simplex uterus and female reproductive system are unique and not well-mimicked by laboratory animal models, limiting translatability to humans. Wide ranges of doses and durations of quercetin treatment were reported in both animal and human studies, albeit with limited or no toxicity. The source(s), purity, and formulation of quercetin are often missing from publications. Quercetin has been administered as a purified product, in combination with other well-defined agents (e.g., NAC), and as a component of a quercetin-containing plant extract or mixture. In these cases, quercetin’s contributions to the observed outcomes are unclear. The poor solubility of quercetin in aqueous solutions, its low bioavailability, unknown tissue and blood/plasma concentrations, and the lack of standardized dosing regimens are other significant concerns. Finally, the small cohorts in most human studies testing quercetin in the setting of uterine conditions further limit their rigor, generalizability, and translatability.

These and other critical gaps prevent advancing quercetin from a potentially ‘promising preclinical candidate’ to an ‘evidence-based therapeutic’ for uterine-related conditions. One issue is safety. The US Food and Drug Administration (FDA) considers quercetin as GRAS or generally recognized as safe. According to WebMD, quercetin may cause headaches or tingling in arms and legs [187]. Heinz et al. published results of a double-blinded, placebo-controlled, randomized trial with females (30–79 years of age) who received oral quercetin supplementation at either 500 mg/day (*n* = 38) or 1000 mg/day (*n* = 40) vs. a placebo (*n* = 42) for 12 weeks, with no adverse effects [188]. Nonetheless, larger and longer-term safety studies of various defined quercetin formulations in diverse populations are needed.

As discussed above, quercetin exhibits variable and often reduced oral bioavailability in humans, which limits its potential therapeutic use [7,9]. Similarly, the limited bioavailability of quercetin in animal models decreases its therapeutic potential. Furthermore, quercetin’s bioavailability and pharmacokinetics differ between various animals and animal models and likely differ dramatically between animal models and humans. Thus, inconsistent quercetin absorption rates and metabolism times would result in varying circulating blood levels and tissue levels in pre-clinical trials, which may lead to inaccurate therapeutic values when applied to humans. This area of research would greatly benefit from standardized sources of quercetin, quercetin formulations, and dosing protocols and consistent pharmacokinetic characterization studies in humans. Pharmacokinetic studies confirm that formulation significantly influences circulating quercetin levels, underscoring the need to develop and evaluate various formulations and enhanced absorption/delivery methods. For example, quercetin purified from *Sophora japonica* and formulated with Phytosome^®^ (also known as Quercefit™ from Indina, Milan, Italy) is a highly available formulation in which quercetin is coated with lecithin to enhance its bioabsorption by at least 10-fold [189]. Also, packaging quercetin in lipid micelles or nanoparticles improves its bioabsorption [8,11] and tissue penetration. Various other methods for improving quercetin’s bioavailability have been described [190]. Of course, along with pharmacokinetic studies, the safety of these formulations needs to be tested in large, diverse populations at multiple doses for short and long durations.

Future clinical investigations of quercetin should incorporate pharmacokinetic and pharmacodynamic assessments, including direct quantification of quercetin and its metabolites in plasma and uterine-relevant compartments (e.g., menstrual effluent or shed endometrium). Emerging approaches, such as menstrual effluent analysis, provide feasible models for uterine-specific biomarker evaluation [57,60,61,89]. Rigorous, randomized, human clinical trials incorporating tissue-level measurements and serum-level assessments of quercetin are needed to establish whether quercetin’s systemic effects translate into meaningful changes in endometrial inflammatory and endocrine signaling pathways. Quercetin should be further studied as a potentially potent, affordable, and accessible therapeutic agent that could improve uterine health and fertility at a time when it is needed most. Additionally, dose and timing of quercetin administration are important considerations. When given to rats for 7 days, low-dose quercetin (10 mg/kg/day) vs. high-dose quercetin (100 mg/kg/day) showed different results on endometrial tissues, with low dose being anti-estrogenic (anti-proliferative, decreased stromal density and thickness) and high dose being pro-estrogenic (pro-proliferative, increased stromal density and endometrial thickness) [191].

Another barrier relates to economics. Despite its broad therapeutic potential, quercetin is unlikely to be the next ‘blockbuster’ treatment, mostly because of profitability issues. Quercetin has been on the ‘supplement market’ for many years. Thus, patents are harder to obtain, and several challenges are imposed by regulatory bodies to develop ‘natural’ quercetin-based treatments, making it less lucrative for companies to invest.

These barriers should not prevent quercetin from becoming a potentially effective therapeutic. Nor should they impede advancements in uterine health. There is hope that investments in women’s health research will set off a chain of positive reactions leading to a self-reinforcing cycle of growth and improvement, offsetting the dismal statistics and deficiency of women’s health-related funding over the last century. A recent National Academies Report proposed several recommendations to facilitate such changes, including the formation of a new women’s health institute (to join the other institutes of the NIH), expansion of oversight and support for women’s health research across all institutes of the NIH, and encouraging Congress to appropriate additional funding for women’s health-specific research [192]. Improved funding would certainly provide impetus and support for transformative uterine health research to significantly advance women’s health.

## 6. Conclusions

Many uterine conditions lack effective treatments. Published evidence reveals quercetin’s pleiotropic effects and suggests that it may be a compelling ‘candidate therapeutic’ for several uterine conditions, including endometrial cancer, endometriosis, adenomyosis, chronic endometritis, uterine fibroids, and PCOS (Table 1, Table 2 and Table 3 and Figure 2). However, more rigorous and robust pre-clinical and clinical studies are required to confirm its therapeutic utility.

NOTE: While this manuscript was under review, PCOS was renamed PMOS (polyendocrine metabolic ovarian syndrome) because PCOS was considered an “inaccurate and misleading term”. The term PMOS better reflects the multisystem health effects and the broad clinical features of this condition, including endocrine alterations (e.g., insulin, androgens, and ovarian hormones), metabolic disorders (e.g., obesity, type 2 diabetes, dyslipidemia, and low-grade inflammation), and reproductive dysfunction (e.g., menstrual cycle abnormalities, infertility, and pregnancy complications) [193].

## Data Availability

No new data were created or analysed in this study. Data sharing is not applicable to this article.

## List of Abbreviations

DHEA	dehydroepiandrosterone
ECM	extracellular matrix
E2	estrogen
ERα	estrogen receptor alpha
ERβ	estrogen receptor beta
eSCs	endometrial stromal cells
FSH	Follicle-stimulating hormone
GLP-1	glucagon-like peptide 1
IGFBP1	insulin growth factor binding protein 1
IP	intraperitoneal
LH	luteinizing hormone
LPS	lipopolysaccharide
LTZ	letrozole
NHPs	non-human primates
P4	progesterone
PAMP	pathogen-associated molecular pattern
PCOS	polycystic ovary syndrome
PGE2	prostaglandin E2
PGR	progesterone receptor
PRL	prolactin
ROS	reactive oxygen species
SASP	senescence-associated secretory phenotype
T	testosterone
TFC	total flavonoids of *Clinopodium chinense*
TP	testosterone propionate
TRPV1	transient receptor potential vanilloid 1
YHHD	Yishen Huatan and Huoxue decoction
